# Chromosome Evolution of the *Liolaemus monticola* (Liolaemidae) Complex: Chromosomal and Molecular Aspects

**DOI:** 10.3390/ani12233372

**Published:** 2022-11-30

**Authors:** Madeleine Lamborot, Carmen Gloria Ossa, Nicolás Aravena-Muñoz, David Véliz, Raúl Araya-Donoso

**Affiliations:** 1 Departamento de Ciencias Ecológicas, Facultad de Ciencias, Universidad de Chile, Santiago 7800003, Chile; 2 Instituto de Biología, Facultad de Ciencias, Universidad de Valparaíso, Valparaíso 2361845, Chile; 3 Centro de Investigación y Gestión de Recursos Naturales, Universidad de Valparaíso, Valparaíso 2361845, Chile; 4 Liceo Alberto Blest Gana, Conchalí 8551270, Chile; 5 Centro de Ecología y Manejo Sustentable de Islas Oceánicas, Coquimbo 1780000, Chile; 6 School of Life Sciences, Arizona State University, Tempe, AZ 85287, USA

**Keywords:** centric fissions, chromosome rearrangements, cytochrome b, population cytogenetics, speciation

## Abstract

**Simple Summary:**

Chromosome variation is highly relevant for evolution because chromosomal mutations can influence speciation. Here, we assessed population cytogenetics in *Liolaemus monticola*, a lizard endemic to Chile that consists of several chromosomal races with highly polymorphic chromosome rearrangements. We sampled individuals from the northernmost distribution of the species to obtain chromosomes and mitochondrial gene sequences and compared the samples to previously published data from other populations across the distribution. Our results show the existence of seven differentiated races of *L. monticola*, each with unique chromosome characteristics and high levels of polymorphism. Interestingly, the geographical delimitation of the races is associated with the presence of rivers that could represent barriers to gene flow. Thus, our study highlights the importance of chromosomal mutations for population differentiation, and in turn, speciation.

**Abstract:**

Chromosomal rearrangements can directly influence population differentiation and speciation. The *Liolaemus monticola* complex in Chile is a unique model consisting of several chromosome races arranged in a latitudinal sequence of increasing karyotype complexity from south to north. Here, we compared chromosomal and mitochondrial cytochrome b data from 15 localities across the northern geographic distribution of *L. monticola.* We expanded the distribution of the previously described Multiple Fissions race (re-described as MF2), in the Coastal range between the Aconcagua River and the Petorca River, and described a new Multiple Fissions 1 (MF1) race in the Andean range. Both races present centric fissions in pairs 1 and 2, as well as a pericentric inversion in one fission product of pair 2 that changes the NOR position. Additionally, we detected a new chromosomal race north of the Petorca River, the Northern Modified 2 (NM2) race, which is polymorphic for novel centric fissions in pairs 3 and 4. Our results increase the number of chromosomal races in *L. monticola* to seven, suggesting a complex evolutionary history of chromosomal rearrangements, population isolation by barriers, and hybridization. These results show the relevant role of chromosome mutations in evolution, especially for highly speciose groups such as *Liolaemus* lizards.

## 1. Introduction

Many closely related plant and animal taxa differ in their chromosomal characteristics [1,2,3,4]. Thus, mutations or chromosomal rearrangements (CR) may play an important role in speciation [5,6,7,8,9,10]. Chromosomal variation (CV) can be mediated by different structural and/or numerical CR, such as Robertsonian translocations (centric fissions and centric fusions), inversions, and translocations. Several models have proposed that CR are causal to genic diversification between populations and therefore facilitate speciation [2,4,9,10,11]. Indeed, CR can usually produce strong evolutionary effects by preventing or reducing the fertility of hybrids, creating a barrier to genetic exchange, and influencing the differentiation of individuals within a population [4,8,9,12,13]. Chromosome variation is of special interest in highly radiated groups, where CR have been proposed as one of the genetic mechanisms associated with high speciation rates, as reported for some reptiles [5,14]. For instance, wide CV has been reported for the pleurodont iguanians *Anolis* [15,16,17], *Sceloporus* [5,14,18,19], and *Liolaemus* [20,21,22,23], and these complex chromosomal rearrangements could be exceptionally relevant for the high speciation described in these groups.

Among iguanians, Liolaemidae is a large and diverse monophyletic family of lizards, endemic to South America, that has been classified into three genera: *Ctenoblepharis*, *Phymaturus*, and *Liolaemus* [24,25,26]. The genus *Liolaemus* is by far the most diverse and highly speciose group in Liolaemidae, with over 292 species widely distributed in southern South America that are characterized by high ecological, chromosomal, genetic, and morphological diversity [23,25]. Thus, the genus *Liolaemus* is a suitable model to study evolutionary biology [23,27,28]. *Liolaemus* shows extensive karyotypic variation, and most taxa karyotyped to date have six pairs of metacentric macrochromosomes and 20–22 microchromosomes (2n = 32–34). The submetacentric chromosome pair 2 has visible secondary constrictions in the long arms, which correspond to the nucleolar organizing region (NOR). These features are considered the ancestral state in *Liolaemus* and primitive among iguanians [6,20,22,23,29,30,31,32]. However, some species present increased chromosome numbers derived from the plesiomorphic karyotypes, which could have originated via Robertsonian rearrangements or polyploidy [23,32].

**Figure 1:**
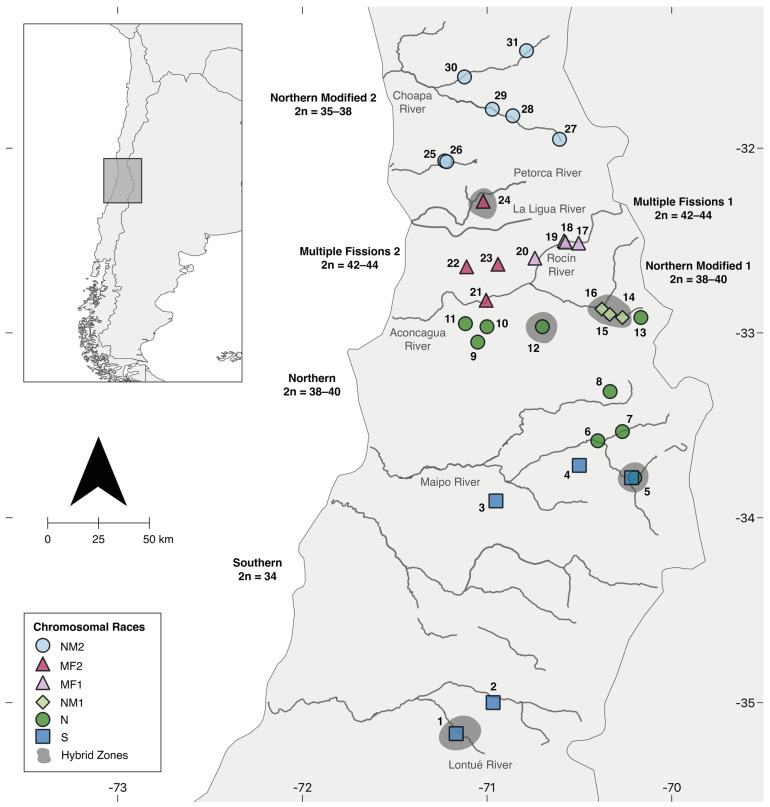
Distribution map of locality samples for six *L. monticola* chromosomal races. Detected potential hybridization zones are shaded. Data include new sampling as well as previously described chromosome races.

One chromosomally derived group in *Liolaemus* is the *L. monticola* complex. This complex is endemic to Chile and is widely distributed throughout the Andes, Coastal, and Transversal Mountain ranges, from 320 to 2000 masl, and between 31° and 35° south latitudes [33]. *L. monticola* is a suitable model to study chromosomal evolution because it displays different CR with elevated polymorphism and diploid chromosome numbers ranging from 2n = 32 to 44 [23]. At least five chromosomal races, whose complexity increases from south to north, have been recognized (Figure 1). The primitive race (P), 2n = 32 (considered ancestral in the *Liolaemus*), is located in the southernmost distribution [34]. The Southern race (S), 2n = 34, is located on the Andes and Coastal Mountain ranges between the Lontué River and the Maipo River [35,36]. The Southern race is characterized by stable fixed translocations between pairs 5 and 7 and one microchromosome pair addition. The Northern race (N), 2n = 38–40, is located from the northern Maipo River and Yeso River to the southern bank of the Aconcagua River [35,36]. The Northern race added centric fissions in pairs 3 and 4, a pericentric inversion in one fission product of chromosome 3, and one pair of microchromosomes. The Northern Modified 1 race (NM1), 2n = 38–40, is located north of the Aconcagua River and east of the Rocín River [37]. The Northern Modified 1 race includes two polymorphisms: an enlarged chromosome in pair 6 and a pericentric inversion in pair 7. Finally, the Multiple Fission race, 2n = 42–44, is found in the “Hierro Viejo” locality between the Aconcagua River and the Petorca River [38]. The Multiple Fission race presents novel polymorphic centric fissions for pairs 1 and 2 and a polymorphic pericentric inversion in one fission product of pair 2.

The patterns of CV between races can be used as genetic markers to improve our understanding of evolution in this species complex. Preliminary studies have shown consistency between the variation detected with mitochondrial *cytb* gene sequences [39], allozymes [40], and morphological analyses [33,41,42] and the south-to-north gradient of chromosomal differentiation. Rivers have been proposed as the main biogeographic barriers that separated and restricted gene flow between the races, especially in association with Pleistocene climatic changes [23,33,34,35,36,37,38,39,40,41,42]. Moreover, chromosomal hybrid zones and introgression between the races have been reported for the *L. monticola* complex in these heterogenous and arid environments [35,36,43,44].

Here, we assessed the patterns of chromosomal and mitochondrial genetic variation in *L. monticola* from fifteen localities northward from the Aconcagua River, which corresponds to the northern geographic distribution of the complex (Figure 1). We complemented our analysis with previously published chromosome data and mitochondrial *cytb* sequences from several populations across the species’ range. Our aim was to infer the underlying processes that explain the patterns of chromosome geographical distribution observed across the *L. monticola* chromosomal races.

## 2. Materials and Methods

### 2.1. Sampling and Data Collection

All *L. monticola* individuals (n = 251) were collected between the spring and fall from 1990 to 2003 (Collecting permit SAG Res: N° 688–3095). We collected lizards from fifteen sites north of the Aconcagua River (Figure 1, Table 1), including the previously published “Hierro Viejo” locality (24) [38], expanding the geographic sampling ~15,000 km^2^. We also added representative localities from the “Southern”, “Northern” [35,36], and “Northern Modified 1” [37] races. Lizards were euthanized using a 0.001 g/g urethane 1% injection in the pineal eye, and tissues were obtained for chromosome analysis and DNA extraction. Voucher specimens were deposited in the collection of the Evolutive Cytogenetic Laboratory, Facultad de Ciencias, Universidad de Chile (CUCH). Catalogue identification and sampling locality coordinates for each karyotyped lizard are listed in Table S1.

### 2.2. Cytogenetic Analyses

Chromosomes were obtained from bone marrow, liver, spleen, and testes using the colchicine-hypotonic pretreated air-drying technique and stained with Giemsa following Lamborot [35]. Selected metaphase plates from each specimen were photographed with a Leitz-Ortholux microscope. Several karyotypes were constructed from enlarged photographs, which were used to score the chromosomal morphology as “genotypes” for the first six macrochromosome complements and the microchromosome pair 7. Chromosome alleles were coded following Lamborot [43]: The ancestral non-fissioned chromosomes were coded “A”, and the metacentric fission rearrangements were coded “B”. Inversions of the ancestral bi-armed chromosomes were coded as “C”, whereas inversions of the fission product in chromosome pair 2 were coded as “D”. The enlarged chromosome, present in some individuals for pair 6, was coded as “E”. Additionally, the novel fission products detected in pairs 3 and 4 from the northernmost locations were coded as “F” and “G”, respectively. Additional observations of spermatocytes at diakinesis, chiasmata, and metaphases II were also made.

The coded genotypes were analyzed in R 4.2.1 [45]. Rogers’ genetic distances [46] were calculated between representative sampling locations using the ‘adegenet’ package [47]. A UPGMA dendrogram was generated based on genetic distances, using the ‘phangorn’ package [48], to assess the relationship between the new sampled sites and previously described chromosomal races. Additionally, we used the genotyped chromosome alleles to determine the population cytogenetic structure with three independent runs of the STRUCTURE v2.3.4 software [49], with a burn-in of 10,000 plus 100,000 MCMC iterations, an admixture ancestry model, and correlated allele frequencies. The most likely number of genetic clusters was estimated with Evanno’s method using the STRUCTURE harvester tool [50]. The UPGMA dendrogram and STRUCTURE suggested the existence of six chromosomal groups within the studied samples. Therefore, further analyses were conducted on these six *L. monticola* chromosomal races. Allele frequencies for each chromosomal pair were calculated for all races using the ‘hierfstat’ package [51]. The allelic richness, expected and observed heterozygosity, and inbreeding coefficient (F_IS_) were also calculated for each race using ‘hierfstat’. A Chi-square test was used to analyze the heterozygote frequencies of each chromosomal pair expected under Hardy–Weinberg equilibrium (HWE) using the ‘pegas’ package [52].

### 2.3. Cytochrome b Sequencing and Analysis

The genomic DNA was obtained from the tissues of 59 individuals across the *L. monticola* distribution (Table S1) following the salt extraction method by Aljanabi & Martínez [53]. Amplification of an 800 bp fragment of the mitochondrial *cytb* gene was performed using GLUDG and CB3 primers [54] under the PCR conditions described by Torres-Pérez et al. [39]. We further added 35 previously published *cytb* sequences from the S, N, NM1, and MF races (Table S1) [39], making a total of 95 sequences representing six chromosomal races. Sequences were visually curated and aligned using the CodonCode Aligner 10.0 (CodonCode Corporation, Dedham, MA, USA).

To assess the phylogenetic relationships between chromosomal races, we performed a Maximum Likelihood phylogenetic inference using RaxML v8 [55] in the CIPRES Science Gateway [56]. A sequence of *L. fuscus* was added as an outgroup. The node support values were obtained from 1000 bootstrap replicates. The trees were visualized using Fig Tree v.1.4.2 [57]. To further explore the mitochondrial relationships between the races, a neighbor-joining haplotype network was constructed using PopART v3 [58]. The F_ST_ index between chromosomal races (based on *cytb* data) was calculated in R using ‘hierfstat’. Additionally, we calculated the number of different haplotypes, the number of segregating sites, haplotype diversity, and nucleotide diversity using the ‘pegas’ package in R. Finally, deviation from neutrality was assessed with a Tajima’s D test for each race using ‘pegas’.

## 3. Results

### 3.1. Patterns of Chromosomal Variation

#### 3.1.1. Andean Range between the Aconcagua River and the Petorca River: The Multiple Fission 1 (MF1) Race, 2n = 42–44

**Figure 2:**
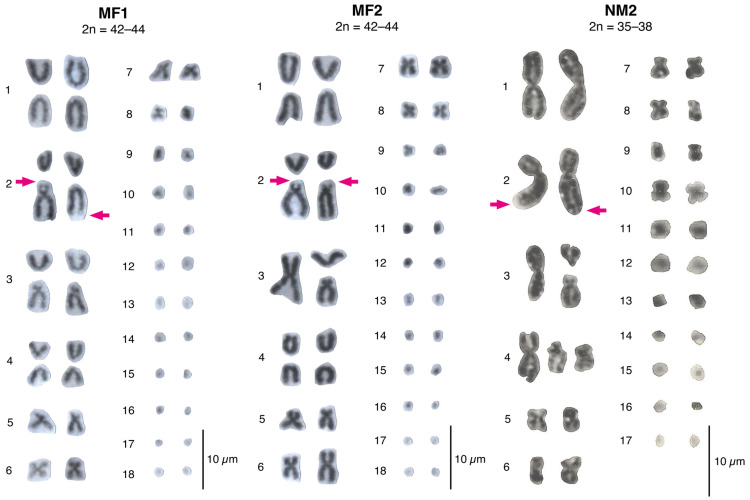
Representative karyotypes for the newly described races of *L. monticola*. Arrow indicates the NOR position on chromosome pair 2. Individuals whose chromosomes are shown are: L2180 (MF1 race), L1362 (MF2 race), and L730 (NM2 race).

**Figure 3:**
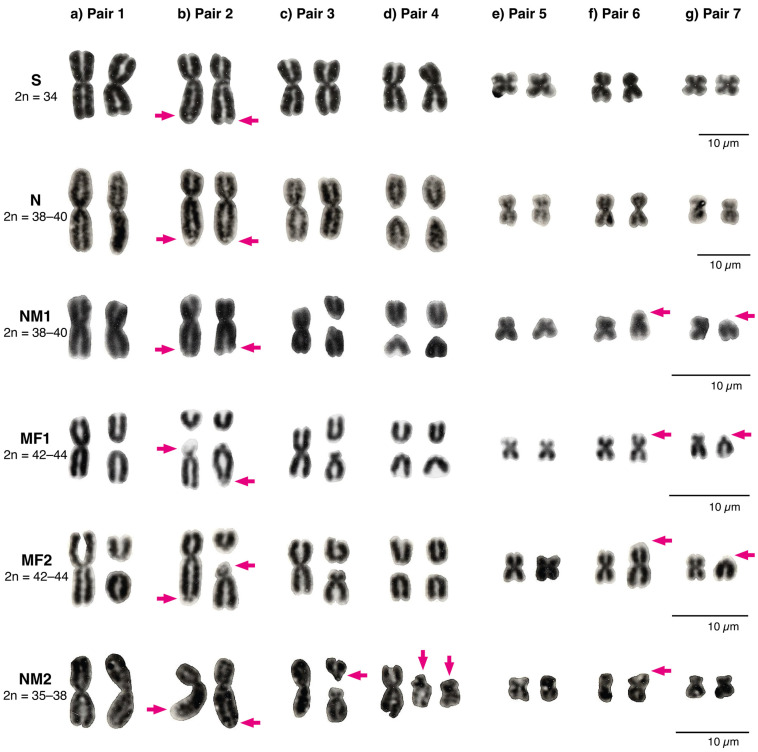
Representation of partial karyotypes for the six macrochromosome pairs (pair 1 to 6) and microchromosome pair 7 of the six chromosome races of *L. monticola*. (**a**) Metacentric pair 1. For the following cases, the arrow depicts; (**b**) the NOR position in pair 2; (**c**) the subacrocentric fission product in pair 3; (**d**) the submetacentric chromosomes in pair 4; (**e**) monomorphic for the submetacentric pair 5; (**f**) the enlarged pair 6; and (**g**) the pericentric inversion in pair 7. Individuals whose chromosomes are shown are: L1085 (S race), L547 (N race), L2651 (NM1 race), L2180 (MF1 race), L1362 (MF2 race), and L730 (NM2 race).

The lizards from four localities (17–20) revealed 39 unique karyotypes (from 54 samples), with a 2n = 42 to 44, 18–20 macrochromosomes, and 24 microchromosomes (Figure 2). Lizards from this area presented polymorphisms for centric fissions on pairs 1 and 2 (Figure 3a,b). Pair 2 fission was highly polymorphic: some individuals presented the NOR at the tip of the long arm of the fission product (genotypes BB and AB), whereas other individuals presented a pericentric inversion in the fission product with the NOR on the tip of the short arm (genotypes AD, BD, and DD). Pair 3 retained the fission polymorphism present in the other races, whereas pair 4 was homozygous for the fission products (Figure 3c,d). The submetacentric pair 5 was homozygous, whereas the enlarged pair 6 and the pericentric inversion of microchromosome pair 7 were polymorphic (Figure 3f,g). In the meiotic diakinesis plates, all the bivalent pairs presented one or two terminal chiasmata, and the trivalent(s) exhibited end-to-end pairing chromosomes (Figure S1).

#### 3.1.2. Coastal Range between the Aconcagua River and the Petorca River: The Multiple Fissions 2 (MF2) Race, 2n = 42–44

The lizards from localities 21–23 and the previously published “Hierro Viejo” (24) exhibited 25 unique karyotypes from 57 individuals, with a 2n = 42 to 44, 18–20 macrochromosomes, and 24 microchromosomes (Figure 2). Similar to the MF1 race, pair 1 showed a centric fission (Figure 3a), which was only polymorphic in the “Hierro Viejo” locality. The pair 2 fission presented the inversion product with the NOR at the tip of the short arm (DD, Figure 3b), and the AD heterozygotes were only present in the “Hierro Viejo”. Pair 3 retained the polymorphism present in the other races (Figure 3c). All lizards were homozygous for the fission of pair 4 and the submetacentric pair 5 (Figure 3d). Pair 6 was polymorphic for an enlarged chromosome, and pair 7 was polymorphic for a pericentric inversion (Figure 3f,g). All bivalent pairs showed one or two terminal chiasmata on the meiotic diakinesis plates, and the trivalent(s) showed end-to-end pairing chromosomes (Figure S1).

#### 3.1.3. Northward from the Petorca River: The Northern Modified 2 Race, 2n = 35–38

In the northernmost locations (25–31), we detected two unique karyotypes from six individuals with a 2n = 35 to 38, 13–16 macrochromosomes, and 22 microchromosomes (Figure 2). The metacentric pairs 1 and 2 were monomorphic (Figure 3a,b). A novel polymorphism was detected for pair 3, in which one of the fission products was acrocentric and the other was submetacentric (Figure 3c). The populations also presented a new polymorphism for pair 4, consisting of two new submetacentric fission products (Figure 3d). All populations were monomorphic for the submetacentric pair 5. The enlarged pair 6 retained the polymorphism, and the metacentric pair 7 was monomorphic (Figure 3f,g). In the diakinesis arrays, pairs 1 and 2 had similar sizes and presented two to three chiasmata per bivalent. The fission pairs 3 and/or 4 of polymorphic lizards showed one or two linear trivalent(s) with two terminal chiasmata each. The bivalents for pairs 5, 6, and 7 presented two terminal chiasmata each (Figure S1).

### 3.2. Population Cytogenetics

**Figure 4:**
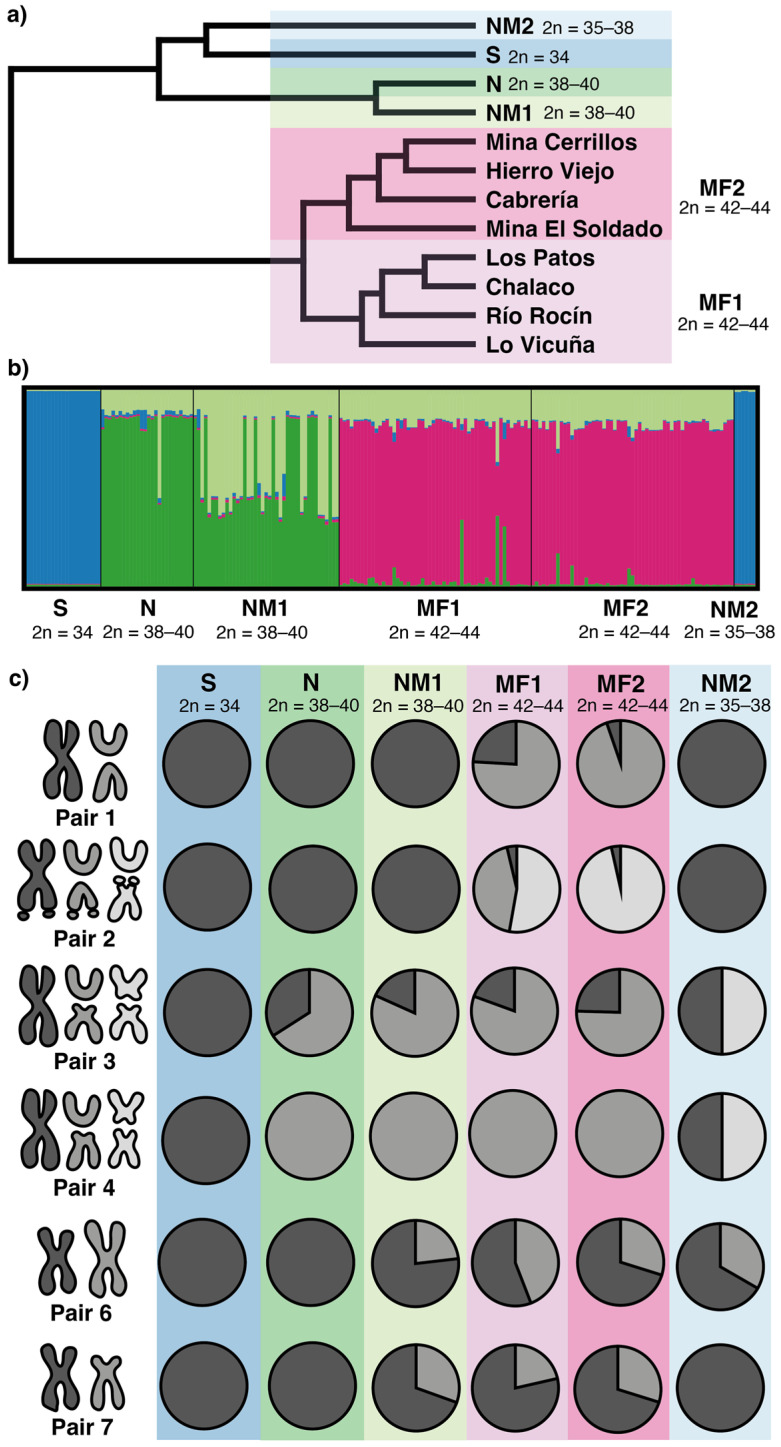
Population cytogenetic analysis of *L. monticola*. (**a**) UPGMA dendrogram based on Rogers’ genetic distances between localities, showing the six chromosomal races organized geographically from left to right. (**b**) Structure plot based on chromosome alleles for K = 4. (**c**) Allele frequencies for each CR of the first seven chromosome pairs in all races.

The UPGMA based on Rogers’ genetic distance differentiated two main groups within *L. monticola* (Figure 4a). In the first group, the northernmost localities of the distribution (NM2) clustered with the Southern race, and they were associated with a second cluster containing the N and NM1 races. A second group included locations from the Multiple Fissions 1 and 2 races and was divided into two clusters corresponding to the Andean (MF1) and Coastal (MF2) ranges, respectively. The structure analysis was consistent with this result, which identified the most likely number of resulting genetic groups as K = 4 (Figure 4b). The first group consisted of locations from the MF1 and MF2 races, the second group included the Southern race plus individuals from the NM2 race, and the other two groups clustered individuals from the N and NM1 races, respectively. Therefore, our results identified six differentiated chromosomal races within the analyzed locations that were distinguished by their geographic distribution, chromosome rearrangements, and genetic-chromosomal population variability. Further analyses were performed based on these six chromosomal races.

Allele frequencies were variable between the populations (Figure 4c). All individuals from the Southern race, 2n = 34, were fixed for the ancestral non-fissioned chromosomes in all pairs. The Northern race, 2n = 38–40, was characterized by a high frequency of the fission polymorphism on pair 3, and the fixation of the pair 4 fission. In the NM1 race, 2n = 38–40, an enlarged pair 6 and a pericentric inversion in pair 7 were characteristic of new rearrangements. The Multiple Fission races were characterized by high-frequency polymorphisms on the fission products of pairs 1 and 2. The main difference between both MF races was that those in the Andean locations (MF1, 2n = 42–44) contained the fission product of pair 2 with and without the inversion (52.77% and 43.52% of frequency, respectively), whereas the inversion of the fission product was almost fixed (96.49% of allele frequency) in the coastal locations (MF2, 2n = 42–44). Finally, the NM2 race, 2n = 35–38, presented a high frequency of the novel rearrangements for pairs 3 and 4, while also sharing the polymorphism for an enlarged pair 6.

The highest allelic richness values were found in both the MF races, whereas the lowest allelic richness values were found in the S race (Table 2). Heterozygosity values were higher in the MF1 and NM2 populations and the lowest in the S race (Table 2). The inbreeding coefficient was significant and positive for the N and NM1 races, whereas it was significant and negative for the NM2 race. We detected significant deviations from the HWE on pairs 3, 4, 6, and 7 (Table 3). Pair 6 had heterozygote deficiency for the NM1 and MF2 races, and pair 7 exhibited heterozygote deficiency in the MF1 and MF2 races. The NM2 race showed heterozygote excess for pairs 3 and 4. The F_ST_ values between chromosomal races were consistent with the UPGMA. The highest F_ST_ values were detected between the S and the rest of the races, whereas the lowest values were detected between the N and NM1 races and between the MF1 and MF2 races (Table 4).

### 3.3. Mitochondrial cytb Sequence Analyses

**Figure 5:**
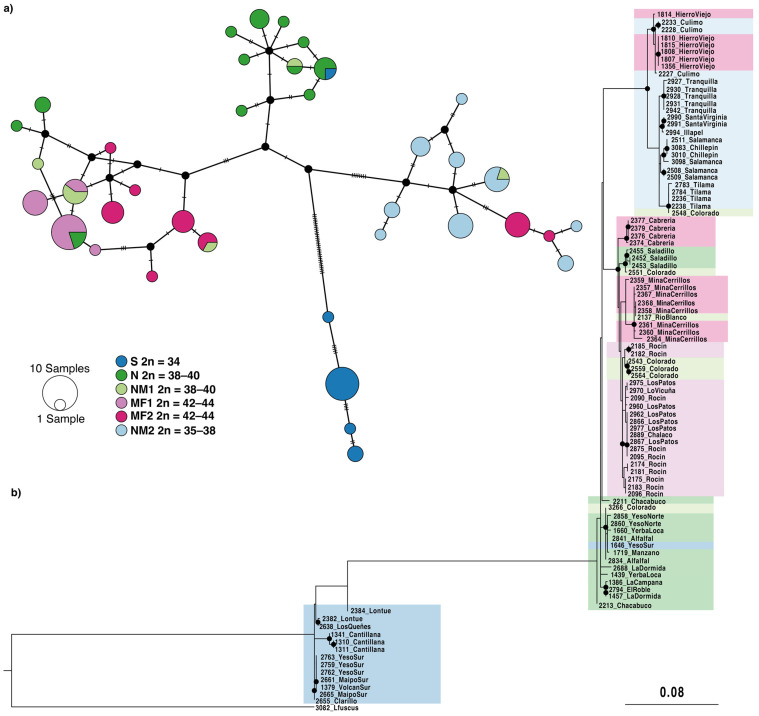
(**a**) Neighbor-joining haplotype network generated from the mitochondrial *cytb* gene with new and previously obtained sequences of *L. monticola.* Each line indicates a mutational step. (**b**) Maximum likelihood phylogenetic reconstruction for the *cytb* gene. Nodes with black filled circles indicate >70% bootstrap support. Note the presence of individuals with discordant positions in both analysis.

Thirty-seven unique haplotypes were detected among the 94 samples of representative *L. monticola* lizards. In both the neighbor-joining network and the maximum likelihood phylogeny, individuals clustered according to their chromosomal race and geographic origin (Figure 5). Nonetheless, the phylogenetic inference had low support values in general. Populations from the S race presented the highest divergence from all the other races in the phylogeny and were separated from the other races by 33 mutational steps in the haplotype network. The NM2 race and the “Hierro Viejo” locality (MF2) formed a supported monophyletic clade in the phylogenetic tree and clustered together in the haplotype network. However, the MF1, MF2, and NM1 races were monophyletically reciprocal to the NM2 and “Hierro Viejo” (Figure 5b). Some lizards presented a discordant position with respect to their chromosomal race in the phylogeny and the haplotype network. For example, the lizards from “Hierro Viejo” (MF2, site 24) clustered with the “Culimó” (NM2, site 25), the samples from “Saladillo” (N, site 13) were grouped with the races MF1 and MF2, and individuals from the NM1 race clustered within different races (Figure 5). Genetic distances (pairwise F_ST_ calculated from *cytb* data) between the races were consistent with geographic distance. F_ST_ values were lower between the NM1, MF1, and MF2 races and higher between the S race and the rest of the races (Table 4). The number of segregating sites was higher in the S and N races, the number of haplotypes and haplotype diversity was higher in the N race, and the nucleotide diversity was higher in the NM1 race, whereas the lowest values were found in the MF1 race. All chromosomal races showed negative values for the Tajima’s D statistic, but the MF1 race was the only race that showed significant deviations from neutrality (Table 5).

## 4. Discussion

This study is the first to analyze and compare individuals across nearly the whole distribution of *L. monticola*. We detected a high intraspecific CV that can be attributed to the concatenation of CR. In addition, we found long-lasting chromosome polymorphisms that can represent the “ghost of hybridization” events or that may be generated after different waves of colonization and recolonization in populations where de novo mutations have occurred. The addition of several samples from the northern geographic distribution increased the total number of chromosomal races to seven (the six analyzed here plus the not included primitive race), which enabled the recognition of two new chromosome races (MF1 and NM2) and the expansion of the previously described “MF” race (now MF2, [38]) from one to various localities. The existence of these races is supported by chromosome and *cytb* gene data and is consistent with previously published cytogenetic, morphological, allozyme, and mitochondrial evidence [35,36,37,38,39,40,41,42,43,44].

### 4.1. Patterns of Chromosomal Variation

#### 4.1.1. The Multiple Fissions Races; MF1 and MF2

The chromosomal data supported the presence of two chromosome races north of the Aconcagua River up to the previously described “Hierro Viejo” locality (17–24) including the MF1, from the Andean Range (17–20), and the MF2, from the Coastal Range (21–24). The MF1 race is geographically and chromosomally intermediate between the NM1 and MF2 races. It retains all chromosome characteristics of the NM1, such as the fixation of the pair 4 fission, polymorphisms for a pair 3 fission, an enlarged pair 6 and a pericentric inversion in chromosome 7, and the same microchromosome number. However, the MF1 race adds three novel chromosomal rearrangements including polymorphic fissions in pairs 1 and 2 and a pericentric inversion in one of the fission products of pair 2 that changes the NOR position (Figure 2 and Figure 3b). On the other hand, the MF2 race at the Coastal Range is considered the most derived of the *L. monticola* complex. It presents the same CR as the MF1 race, but it fixates the fissioned pair 1 (BB) and the fissioned pair 2 with the pericentric inversion (DD; Figure 2 and 3b) in all localities except for the unique “Hierro Viejo” population (see below).

The rearrangements of pair 2 associated with the NOR position constitute a very interesting feature of the Multiple Fissions races. All other races present chromosome 2 as submetacentric (AA) with the NOR at the tip of the long arm. The existence of different chromosome combinations (particularly AB, AD, and BD heterozygotes in the MF1 race), suggests that both chromosomal mutations were sequentially independent events. In this scenario, the pericentric inversion could have followed the centric fission (Figure S2), as described by Kolnicki [59], according to Todd’s karyotypic fission theory, and it could be fixated by meiotic selection stabilizing the karyotype (e.g., mammals [60]). In *L. monticola*, we detected intermediate stages that show the transition from the ancestral pair 2 in the MF1 race to the derived form in the MF2 race. The detection of sequential chromosome mutations is usually not possible, as intermediate CR may remain in low frequency or be lost in natural populations. Porter and Sites [61] and Goyenechea et al. [62] reported similar variability patterns in pair 2 associated with the NOR position in *Sceloporus grammicus*. The *L. monticola* chromosome 2 evolution described here highlights the evolutionary importance of pericentric inversions to stabilize other chromosome rearrangements.

#### 4.1.2. The Northern Modified 2 Race

Lizards from the northernmost distribution of the *L. monticola* complex (25–31) were assigned to a new NM2 race, 2n = 35–38, which included two novel fissions in pairs 3 and 4. Pair 3 was polymorphic in most lizards with a metacentric chromosome and two fission products, one acrocentric and the other submetacentric. This rearrangement is different from the acrocentric and subacrocentric fission products described for pair 3 in the other races (N, NM1, MF1, and MF2) [35,36,37,38]. Pair 4 also presented a unique polymorphism, including a metacentric chromosome and two submetacentric fission products, instead of the common acrocentric fission products detected in pair 4 for the other races (except for the polymorphism found in hybrids between the Southern, 2n = 34, and Northern, 2n = 38–40, races [36,44]). Interestingly, this race shared a similar karyotype with the Southern race (Figure 2 and Figure 3) including a metacentric pair 1, a submetacentric pair 2, a metacentric pair 7, and 22 microcromosomes. This observation contrasts with the expected pattern of increased karyotype complexity from south to north. Furthermore, bivalents 1 and 2 in the NM2 race presented two to three chiasmata (Figure S1). The other races (N, NM1, MF1, and MF2) only present terminal chiasmata, except for the Southern race which shows several chiasmata in bivalents 1 and 2, as observed in the NM2 race [35,36].

### 4.2. Chromosome Polymorphism

We reported strikingly high chromosomal polymorphism levels across the *L. monticola* races (Figure 4, Table 2), which are comparable to the high CV and multiple chromosome races described in *Sceloporus* [61]. The fact that several polymorphisms have persisted in *L. monticola* in high frequency and in conformance with the HWE (Figure 4c, Table 3) suggests that these rearrangements may not be selectively disadvantageous for centric fission heterozygotes compared to homozygotes. For instance, Lamborot and Alvarez Sarret [36] demonstrated that the polymorphism in pair 3 from the Northern race does not appear to undergo abnormal meiotic segregation. In previous reports, the degree of polymorphism of pair 3 varies depending on the genetic background and its geographical origin [34,35,37,42]. In addition, the number of aneuploidies observed in metaphase II may be normal at the Andes Range (less than 5%), 10–23% at the Coast Range, and 26–32% for chromosome hybrids from the hybrid zone [36].

### 4.3. Riverine Barriers and Gene Flow

The species formation process requires the disruption of gene flow by geographic isolation of a panmictic population into two or more populations, thus allowing for the accrual of mutations. Therefore, limited gene flow can differentiate populations and originate races within a species. Both chromosome and mitochondrial *cytb* data supported the existence of differentiated races within *L. monticola*. These populations and races are spatially fragmented and could be considered a metapopulation system [63]. Interestingly, the geographic limits of chromosome races match the presence of rivers throughout Chile. The relevance of riverine barriers for gene flow restriction has been proposed for various taxa (e.g., [64]). Rivers in this area seem to be major barriers to gene flow. Indeed, the Maipo River and the Aconcagua River are proposed barriers to gene flow for *L. monticola* and other reptile species [33,41,42,65,66]. Our analysis showed deep mitochondrial *cytb* divergence between the Southern race and the other chromosome races (Figure 5, Table 4), as reported by Torres-Pérez et al. [39,67]. This result suggests that populations of *L. monticola* may have been geographically isolated for a long period of time, which could even correspond to incipient speciation. 

Geological data for the middle Chilean Andes demonstrate that Pleistocene glaciations were extensive and that rivers could have been larger during past glaciation/deglaciation cycles [68,69,70]. In the last glaciation episodes, the glaciers on the transversal valleys (such as the Maipo Valley, Aconcagua Valley, and Petorca Valley) were particularly well developed. These glacial tongues in central Chile could have interrupted gene flow between chromosomal races prior to the development of rivers. Heusser [71] indicated that the Coastal Range was in general not influenced by glaciers, therefore populations may have found refuge in coastal locations and then recolonized the Andean locations. Our *cytb* results support this possibility by showing signatures of population expansion for all races (Table 5), consistent with Torres-Perez et al. [39]. Therefore, we propose that *L. monticola* had a complex evolutionary history, with rivers (such as the Petorca River, La Ligua River, Aconcagua River, and Maipo River) acting as barriers to gene flow and the Pleistocene glaciation cycles affecting the populations’ evolutionary dynamics.

### 4.4. Hybridization between Chromosomal Races

Discordances were detected in our analyses, as some individuals showed *cytb* sequences related to other chromosome races (Figure 5). This pattern could be associated with introgression, retention of ancestral polymorphisms, or incomplete lineage sorting [65,72]. Parapatry in narrow secondary contact zones and hybridization have previously been proposed for this complex between the chromosomal races: P × S, S × N, N × NM1 (Figure 1). For example, localities nearby the Yeso River (5) constitute a hybrid zone between the Southern and Northern races [35]. The NM1 race, located between two tributaries of the Aconcagua River (Colorado River and the Juncal River, 14–16), was proposed to have a primary hybrid origin [37,40], and the “Chacabuco” (12) was described as a potential hybrid zone between the Coastal and Andean populations within the N race [43]. Thus, it is possible that individuals from different races can produce viable offspring, especially given some potential mechanisms that could stabilize meiosis and maintain stable recombination rates in hybrids [35].

The previously described “Hierro Viejo” population (24) [38], the northernmost population of the MF2 race, shared similar mitochondrial *cytb* sequences with individuals from the “Culimó” (25, NM2). This location can be considered unique among the other MF2 populations because it was the only population that presented AB heterozygotes (5 out of 29 lizards) for the pair 1 fission and AD heterozygotes (4 out of 29 lizards) for the fission and inversion products of pair 2 (both CR are fixed in the other MF2 populations; Figure 4c, Table S1). These polymorphisms could have been retained when diverging from the MF1 race. Alternatively, this karyotype and the discordant mitochondrial relationships for this population could be indicative of introgression between the MF2 and NM2 races, which at this location are only ~30 km apart but are separated by the Petorca River. One less plausible hypothesis is that these CR correspond to de novo mutations.

### 4.5. Model of Chromosome Evolution

We propose a sequence of events describing the chromosomal rearrangements occurring through the evolution of *L. monticola* races based on our results. Initially, individuals with a karyotype similar to the Southern race, 2n = 34, could have distributed throughout the entire geographic range of the complex. A first colonization event could have originated the Northern race, 2n = 38–40, with its characteristic rearrangements (homozygotic centric fission of pair 4 and centric fission polymorphism in pair 3), expanding northward from the Maipo River [35,36]. The Northern Modified 1 race, 2n = 38–40, probably originated from the Andean range of the N race, with a polymorphism for an enlarged chromosome 6 and a polymorphic pericentric inversion in chromosome 7 [37]. Then, the NM1 race gave rise to the Multiple Fissions 1 race, 2n = 42–44, with its new polymorphisms for fissions in pairs 1 and 2. Subsequently, the inversion product of the pair 2 was fixed (DD homozygotes) in the MF2 race stabilizing the inversion–noninversion heterozygote, as described above. To explain the unique rearrangements in the NM2 race, 2n = 35–38, and its similarity to the S race, we propose an independent process of colonization in which the ancient “Southern like” populations underwent centric fissions at the northernmost range of the complex distribution. Chromosome evidence supports the hypothesis of a colonization initiated by the Southern race that originated the other chromosomally derived races, which continued with processes of colonization, hybridization, and replacement [23]. This is consistent with morphological [33,41,42], alloenzyme [40], and mitochondrial genetic data [39,67].

The sequential chromosome rearrangements that originated all races, and the latitudinally arranged karyotypic variation (corroborated by the increased complexity from south to north, except for the NM2 race), resemble Hall’s “cascade model of speciation” [14], the “chain process” [2], and the “primary chromosomal allopatry” [4] hypotheses, among others. Similar processes have been reported for chromosomal speciation of *Sceloporus* lizards [18,19], *Sorex* shrews [73], and *Ctenomys* rodents [74]. Geological complexity such as rivers, transversal mountain ranges, and latitudinal climatic gradients, may have also played an important role in restricting gene flow and promoting race differentiation. Moreover, when considering other *Liolaemus* species, there is a correlation between the chromosome number and environmental gradients where the number of centric fissions increases towards more arid and heterogeneous environments in northern Chile [21,32]. This highlights the relevance of understanding the adaptive potential of the Robertsonian-centric fissions for colonizing more challenging environments.

## 5. Conclusions

Our results present a unique opportunity to investigate incipient in situ evolution, recognizing the chromosome rearrangements that account for the different races found in *L. monticola* throughout its distribution. Further studies would help to unravel the mechanisms associated with the origin and fixation of these chromosome mutations in this complex, as well as their implications on fitness and population differentiation. Here, we have highlighted the importance of chromosomal rearrangements for the evolution and potential speciation in highly radiated groups such as *Liolaemus* lizards.

## Figures and Tables

**Table 1:** Sampling locations for the different races of *L. monticola*. See Table S1 for a full list of sampled individuals.

Race	Location	Race	Location
Southern, 2n = 34	1. Lontué	Northern Modified 1, 2n = 38–40	16. Colorado River North
	2. Los Queñes	Multiple Fissions 1, 2n = 42–44	17. Rocín River
	3. Cantillana		18. Los Patos
	4. Clarillo River		19. Chalaco
	5. Yeso River South		20. Lo Vicuña
Northern, 2n = 38–40	5. Yeso River North	Multiple Fissions 2, 2n = 42–44	21. Cabrería
	6. El Manzano		22. El Soldado
	7. El Alfalfal		23. Mina Cerrillos
	8. Yerba Loca		24. Hierro Viejo
	9. El Roble	Northern Modified 2, 2n = 35–38	25. Culimó
	10. La Dormida		26. Tilama
	11. La Campana		27. Tranquilla
	12. Chacabuco		28. Chillepín
	13. Saladillo		29. Salamanca
Northern Modified 1, 2n = 38–40	14. Blanco River		30. Illapel
	15. Colorado River South		31. Santa Virginia

**Table 2:** Chromosome variability parameters in six populations of *L. monticola* (S: Southern; N: Northern; NM1: Northern Modified 1; MF1: Multiple Fissions 1; MF2: Multiple Fissions 2; NM2: Northern Modified 2). Allele richness (Ar), observed (Ho) and expected (He) heterozygosity, and inbreeding coefficient (F_IS_), * *p* < 0.05.

	S	N	NM1	MF1	MF2	NM2
Ar	1	1.142	1.412	1.749	1.544	1.429
Ho	0	0.060	0.115	0.273	0.155	0.381
He	0	0.064	0.157	0.294	0.199	0.210
F_IS_	-	0.058 *	0.273 *	0.078	0.126	−0.810 *

**Table 3:** Chi squared test *p*-values for Hardy–Weinberg equilibrium for the first seven chromosome pairs from each *L. monticola* chromosomal race (S: Southern; N: Northern; NM1: Northern Modified 1; MF1: Multiple Fissions 1; MF2: Multiple Fissions 2; NM2: Northern Modified 2). * *p* < 0.05.

	S	N	NM1	MF1	MF2	NM2
Pair 1	1	1	1	0.113	0.675	1
Pair 2	1	1	1	0.838	0.784	1
Pair 3	1	0.844	0.089	0.971	0.081	0.014 *
Pair 4	1	1	1	1	1	0.014 *
Pair 5	1	1	1	1	1	1
Pair 6	1	1	0.014 *	0.542	0.002 *	0.221
Pair 7	1	1	0.381	0.001 *	<0.001 *	1

**Table 4:** Differentiation between the six *Liolaemus monticola* chromosomal races (S: Southern; N: Northern; NM1: Northern Modified 1; MF1: Multiple Fissions 1; MF2: Multiple Fissions 2; NM2: Northern Modified 2). Above the diagonal, F_ST_ values obtained from chromosome “alleles”; below the diagonal *cytb* sequences data *: *p* < 0.05.

	S	N	NM1	MF1	MF2	NM2
**S**	-	0.853 *	0.712 *	0.680 *	0.777 *	0.653 *
**N**	0.332 *	-	0.151 *	0.497 *	0.645 *	0.652 *
**NM1**	0.355 *	0.036 *	-	0.443 *	0.590	0.530
**MF1**	0.422 *	0.176 *	0.073 *	-	0.123 *	0.552 *
**MF2**	0.351 *	0.078 *	0.028 *	0.159 *	-	0.684 *
**NM2**	0.349 *	0.228 *	0.194 *	0.380 *	0.167 *	-

**Table 5:** Genetic variability of *cytb* in the *Liolaemus monticola* chromosome race samples (S: Southern; N: Northern; NM1: Northern Modified 1; MF1: Multiple Fissions 1; MF2: Multiple Fissions 2; NM2: Northern Modified 2). Sample size (n), number of haplotypes (K), number of segregating sites (S), haplotype diversity (H), nucleotide diversity (π), and Tajima’s D statistic for each *L. monticola* chromosomal race. *: *p* < 0.05.

	S	N	NM1	MF1	MF2	NM2
n	14	20	7	16	19	19
K	8	16	6	7	10	13
S	91	68	56	12	52	38
H	0.824	0.979	0.952	0.867	0.895	0.947
π	0.025	0.022	0.028	0.003	0.026	0.012
D	−1.949	−1.071	−1.086	−2.403 *	−0.731	−0.837

## Data Availability

Data used for this study and Genebank accession numbers are available in the Supplementary Material.

## Supplementary Materials

The following supporting information can be downloaded at: https://www.mdpi.com/article/10.3390/ani12233372/s1, Table S1. Full data for the sampled individuals. Figure S1. Representative meiotic karyotype for the first seven chromosome pairs for lizards from the newly described chromosomal races of *Liolaemus monticola*. Figure S2. Model of evolution for the chromosome pair 2 in *Liolaemus monticola* in the Multiple Fission races.
