# Supplementary material for: Chromosome Evolution of the Liolaemus monticola (Liolaemidae) Complex: Chromosomal and Molecular Aspects
Source: Animals (Basel). 2022 Nov 30;12(23):3372. doi: 10.3390/ani12233372 (PMC9737244; doi:10.3390/ani12233372)
Supplement: Supplementary file 1 [file animals-12-03372-s001.zip › animals-2033429-supplementary.pdf]

Electronic Supplementary Material for…

Chromosome Evolution of the *Liolaemus monticola* (Liolaemidae) Complex: Chromosomal and Molecular Aspects

Madeleine Lamborot, Carmen Gloria Ossa, Nicolás Aravena-Muñoz, David Véliz and Raúl Araya-Donoso

Supplementary Tables

Table S1. Full data for the sampled individuals. Unique identifier in the collection of the Evolutive Cytogenetic Laboratory (CUCH), sampling locality and coordinates, corresponding chromosome race: Southern (S), Northern (N), Northern Modified 1 (NM1), Multiple Fissions 1 (MF1), Multiple Fissions 2 (MF2) and Northern Modified 2 (NM2). Genebank accession numbers for mitochondrial *cytb* sequences, and chromosome alleles for first six macrochromosome pairs and microchromosome pair 7.

ID	Species	Locality	Latitude	Longitude	Race	Genebank Accession Number	P1	P2	P3	P4	P5	P6	P7
727	*Liolaemus monticola*	Salamanca	-31.788889	-70.971111	NM2	-	AA	AA	AF	AG	AA	AE	AA
730	*Liolaemus monticola*	Salamanca	-31.788889	-70.971111	NM2	-	AA	AA	AF	AG	AA	AE	AA
817	*Liolaemus monticola*	Salamanca	-31.788889	-70.971111	NM2	-	AA	AA	AF	AG	AA	AA	AA
823	*Liolaemus monticola*	Salamanca	-31.788889	-70.971111	NM2	-	AA	AA	AF	AG	AA	AA	AA
1310	*Liolaemus monticola*	Cantillana	-33.909052	-70.951620	S	AY851735	-	-	-	-	-	-	-
1311	*Liolaemus monticola*	Cantillana	-33.909052	-70.951620	S	AY851736	-	-	-	-	-	-	-
1341	*Liolaemus monticola*	Cantillana	-33.909052	-70.951620	S	OP886909	-	-	-	-	-	-	-
1354	*Liolaemus monticola*	Hierro Viejo	-32.287500	-71.020556	MF2	-	BB	DD	BB	BB	AA	EE	AA
1356	*Liolaemus monticola*	Hierro Viejo	-32.287500	-71.020556	MF2	OP886911	AB	DD	BB	BB	AA	AE	AA
1357	*Liolaemus monticola*	Hierro Viejo	-32.287500	-71.020556	MF2	-	BB	DD	AB	BB	AA	EE	AC
1358	*Liolaemus monticola*	Hierro Viejo	-32.287500	-71.020556	MF2	-	BB	DD	BB	BB	AA	AA	AA
1359	*Liolaemus monticola*	Hierro Viejo	-32.287500	-71.020556	MF2	-	BB	DD	AB	BB	AA	EE	CC
1360	*Liolaemus monticola*	Hierro Viejo	-32.287500	-71.020556	MF2	-	BB	DD	AB	BB	AA	AA	AA
1361	*Liolaemus monticola*	Hierro Viejo	-32.287500	-71.020556	MF2	-	BB	DD	BB	BB	AA	AA	AA
1362	*Liolaemus monticola*	Hierro Viejo	-32.287500	-71.020556	MF2	-	AB	AD	BB	BB	AA	AE	AC
1379	*Liolaemus monticola*	Volcan Sur	-33.800000	-70.150000	S	AY851734	-	-	-	-	-	-	-
1386	*Liolaemus monticola*	La Campana	-32.950000	-71.116667	N	AY851715	-	-	-	-	-	-	-
1439	*Liolaemus monticola*	Yerba Loca	-33.316667	-70.333333	N	AY850618	-	-	-	-	-	-	-
1457	*Liolaemus monticola*	La Dormida	-33.050000	-71.050000	N	AY851726	-	-	-	-	-	-	-
1459	*Liolaemus monticola*	Los Queñes	-35.000000	-70.966667	S	-	AA	AA	AA	AA	AA	AA	AA
1460	*Liolaemus monticola*	Los Queñes	-35.000000	-70.966667	S	-	AA	AA	AA	AA	AA	AA	AA
1464	*Liolaemus monticola*	Los Queñes	-35.000000	-70.966667	S	-	AA	AA	AA	AA	AA	AA	AA
1608	*Liolaemus monticola*	Hierro Viejo	-32.287500	-71.020556	MF2	-	BB	DD	BB	BB	AA	AA	AA
1609	*Liolaemus monticola*	Hierro Viejo	-32.287500	-71.020556	MF2	-	BB	DD	AB	BB	AA	AA	AA
1610	*Liolaemus monticola*	Hierro Viejo	-32.287500	-71.020556	MF2	-	BB	DD	AB	BB	AA	AA	AA
1612	*Liolaemus monticola*	Hierro Viejo	-32.287500	-71.020556	MF2	-	BB	AD	BB	BB	AA	AA	AC
1614	*Liolaemus monticola*	Hierro Viejo	-32.287500	-71.020556	MF2	-	BB	DD	BB	BB	AA	AE	CC
1616	*Liolaemus monticola*	Hierro Viejo	-32.287500	-71.020556	MF2	-	BB	DD	AB	BB	AA	EE	AC

1617	*Liolaemus monticola*	Hierro Viejo	-32.287500	-71.020556	MF2	-	BB	DD	BB	BB	AA	AE	AC
1618	*Liolaemus monticola*	Hierro Viejo	-32.287500	-71.020556	MF2	-	AB	DD	AB	BB	AA	AA	AA
1619	*Liolaemus monticola*	Hierro Viejo	-32.287500	-71.020556	MF2	-	BB	DD	AB	BB	AA	AA	AA
1620	*Liolaemus monticola*	Hierro Viejo	-32.287500	-71.020556	MF2	-	BB	DD	BB	BB	AA	AA	AA
1646	*Liolaemus monticola*	Yeso Sur	-33.783333	-70.216667	S	AY850620	-	-	-	-	-	-	-
1660	*Liolaemus monticola*	Yerba Loca	-33.316667	-70.333333	N	AY850619	-	-	-	-	-	-	-
1719	*Liolaemus monticola*	El Manzano	-33.583333	-70.400000	N	AY850617	-	-	-	-	-	-	-
1807	*Liolaemus monticola*	Hierro Viejo	-32.287500	-71.020556	MF2	AY851705	AB	DD	BB	BB	AA	EE	AA
1808	*Liolaemus monticola*	Hierro Viejo	-32.287500	-71.020556	MF2	AY851704	BB	DD	BB	BB	AA	AA	AA
1809	*Liolaemus monticola*	Hierro Viejo	-32.287500	-71.020556	MF2	-	BB	DD	BB	BB	AA	AE	AC
1810	*Liolaemus monticola*	Hierro Viejo	-32.287500	-71.020556	MF2	OP886912	AB	DD	BB	BB	AA	EE	AA
1811	*Liolaemus monticola*	Hierro Viejo	-32.287500	-71.020556	MF2	-	BB	DD	AB	BB	AA	EE	AA
1812	*Liolaemus monticola*	Hierro Viejo	-32.287500	-71.020556	MF2	-	BB	DD	AB	BB	AA	AE	CC
1814	*Liolaemus monticola*	Hierro Viejo	-32.287500	-71.020556	MF2	OP886915	BB	DD	BB	BB	AA	AE	AA
1815	*Liolaemus monticola*	Hierro Viejo	-32.287500	-71.020556	MF2	OP886910	BB	DD	BB	BB	AA	AA	AA
1816	*Liolaemus monticola*	Hierro Viejo	-32.287500	-71.020556	MF2	-	BB	DD	BB	BB	AA	AE	AA
1817	*Liolaemus monticola*	Hierro Viejo	-32.287500	-71.020556	MF2	-	BB	AD	AB	BB	AA	AE	AA
1818	*Liolaemus monticola*	Hierro Viejo	-32.287500	-71.020556	MF2	-	BB	AD	BB	BB	AA	EE	AC
1862	*Liolaemus monticola*	El Soldado	-32.642222	-71.110278	MF2	-	BB	DD	AB	BB	AA	AA	AC
1863	*Liolaemus monticola*	El Soldado	-32.642222	-71.110278	MF2	-	BB	DD	AB	BB	AA	AA	AA
1864	*Liolaemus monticola*	El Soldado	-32.642222	-71.110278	MF2	-	BB	DD	AB	BB	AA	AA	CC
1865	*Liolaemus monticola*	El Soldado	-32.642222	-71.110278	MF2	-	BB	DD	BB	BB	AA	AA	AC
1866	*Liolaemus monticola*	El Soldado	-32.642222	-71.110278	MF2	-	BB	DD	AB	BB	AA	AA	CC
1867	*Liolaemus monticola*	El Soldado	-32.642222	-71.110278	MF2	-	BB	DD	AB	BB	AA	AA	CC
1868	*Liolaemus monticola*	El Soldado	-32.642222	-71.110278	MF2	-	BB	DD	BB	BB	AA	AA	CC
1869	*Liolaemus monticola*	El Soldado	-32.642222	-71.110278	MF2	-	BB	DD	BB	BB	AA	AA	AC
1870	*Liolaemus monticola*	El Soldado	-32.642222	-71.110278	MF2	-	BB	DD	BB	BB	AA	AE	AA
1871	*Liolaemus monticola*	El Soldado	-32.642222	-71.110278	MF2	-	BB	DD	AB	BB	AA	AA	AA
2090	*Liolaemus monticola*	Rocín	-32.513611	-70.503611	MF1	OP886957	BB	BD	BB	BB	AA	AE	AA
2091	*Liolaemus monticola*	Rocín	-32.513611	-70.503611	MF1	-	AB	DD	BB	BB	AA	EE	AA
2092	*Liolaemus monticola*	Rocín	-32.513611	-70.503611	MF1	-	BB	BD	BB	BB	AA	AA	AA
2093	*Liolaemus monticola*	Rocín	-32.513611	-70.503611	MF1	-	AB	BD	BB	BB	AA	AA	AC
2095	*Liolaemus monticola*	Rocín	-32.513611	-70.503611	MF1	OP886963	AB	BB	BB	BB	AA	AE	AA
2096	*Liolaemus monticola*	Rocín	-32.513611	-70.503611	MF1	OP886952	AB	BD	BB	BB	AA	EE	AA
2097	*Liolaemus monticola*	Rocín	-32.513611	-70.503611	MF1	-	AB	BB	BB	BB	AA	EE	AA
2098	*Liolaemus monticola*	Rocín	-32.513611	-70.503611	MF1	-	BB	BD	AB	BB	AA	AE	AA
2099	*Liolaemus monticola*	Rocín	-32.513611	-70.503611	MF1	-	AB	BB	BB	BB	AA	AA	AA
2100	*Liolaemus monticola*	Rocín	-32.513611	-70.503611	MF1	-	AB	DD	AB	BB	AA	AA	AA
2108	*Liolaemus monticola*	Lontué	-35.166667	-71.166667	S	-	AA	AA	AA	AA	AA	AA	AA
2109	*Liolaemus monticola*	Lontué	-35.166667	-71.166667	S	-	AA	AA	AA	AA	AA	AA	AA
2137	*Liolaemus monticola*	Río Blanco	-32.916667	-70.266667	NM1	AY851712	-	-	-	-	-	-	-
2170	*Liolaemus monticola*	Chalaco	-32.501111	-70.582778	MF1	-	AB	DD	AA	BB	AA	EE	AC
2171	*Liolaemus monticola*	Chalaco	-32.501111	-70.582778	MF1	-	BB	BD	BB	BB	AA	EE	AC
2172	*Liolaemus monticola*	Chalaco	-32.501111	-70.582778	MF1	-	AB	DD	BB	BB	AA	AE	AA
2173	*Liolaemus monticola*	Chalaco	-32.501111	-70.582778	MF1	-	BB	BB	AB	BB	AA	EE	AA
2174	*Liolaemus monticola*	Rocín	-32.513611	-70.503611	MF1	OP886950	BB	BD	BB	BB	AA	EE	AC
2175	*Liolaemus monticola*	Rocín	-32.513611	-70.503611	MF1	OP886953	BB	AB	AB	BB	AA	AA	AC
2176	*Liolaemus monticola*	Rocín	-32.513611	-70.503611	MF1	-	AB	BB	BB	BB	AA	AA	AA
2177	*Liolaemus monticola*	Rocín	-32.513611	-70.503611	MF1	-	AB	BD	AA	BB	AA	AE	AA
2178	*Liolaemus monticola*	Rocín	-32.513611	-70.503611	MF1	-	BB	BD	BB	BB	AA	AE	AA
2180	*Liolaemus monticola*	Rocín	-32.513611	-70.503611	MF1	-	AB	BD	AB	BB	AA	AE	AC
2181	*Liolaemus monticola*	Rocín	-32.513611	-70.503611	MF1	OP886951	BB	BB	BB	BB	AA	EE	CC
2182	*Liolaemus monticola*	Rocín	-32.513611	-70.503611	MF1	OP886948	BB	BB	BB	BB	AA	AE	CC
2183	*Liolaemus monticola*	Rocín	-32.513611	-70.503611	MF1	OP886954	BB	BD	BB	BB	AA	AA	AA
2184	*Liolaemus monticola*	Rocín	-32.513611	-70.503611	MF1	-	AB	BB	BB	BB	AA	AA	AA
2185	*Liolaemus monticola*	Rocín	-32.513611	-70.503611	MF1	OP886949	BB	BB	BB	BB	AA	AA	CC
2211	*Liolaemus monticola*	Chacabuco	-32.966667	-70.700000	N	AY851719	-	-	-	-	-	-	-
2213	*Liolaemus monticola*	Chacabuco	-32.966667	-70.700000	N	AY851718	-	-	-	-	-	-	-
2227	*Liolaemus monticola*	Culimó	-32.069167	-71.228611	NM2	OP886916	-	-	-	-	-	-	-

2228	*Liolaemus monticola*	Culimó	-32.069167	-71.228611	NM2	OP886913	-	-	-	-	-	-	-
2233	*Liolaemus monticola*	Culimó	-32.069167	-71.228611	NM2	OP886914	-	-	-	-	-	-	-
2236	*Liolaemus monticola*	Tilama	-32.072778	-71.218056	NM2	OP886920	-	-	-	-	-	-	-
2238	*Liolaemus monticola*	Tilama	-32.072778	-71.218056	NM2	OP886918	-	-	-	-	-	-	-
2286	*Liolaemus monticola*	Saladillo	-32.916667	-70.166667	N	-	AA	AA	AA	BB	AA	AA	AA
2287	*Liolaemus monticola*	Saladillo	-32.916667	-70.166667	N	-	AA	AA	BB	BB	AA	AA	AA
2288	*Liolaemus monticola*	Saladillo	-32.916667	-70.166667	N	-	AA	AA	BB	BB	AA	AA	AA
2289	*Liolaemus monticola*	Saladillo	-32.916667	-70.166667	N	-	AA	AA	AB	BB	AA	AA	AA
2290	*Liolaemus monticola*	Saladillo	-32.916667	-70.166667	N	-	AA	AA	BB	BB	AA	AA	AA
2357	*Liolaemus monticola*	Mina Cerrillos	-32.628333	-70.940278	MF2	OP886943	BB	DD	AB	BB	AA	AA	CC
2358	*Liolaemus monticola*	Mina Cerrillos	-32.628333	-70.940278	MF2	AY851706	BB	DD	AB	BB	AA	AE	AA
2359	*Liolaemus monticola*	Mina Cerrillos	-32.628333	-70.940278	MF2	AY851707	BB	DD	BB	BB	AA	AA	AA
2360	*Liolaemus monticola*	Mina Cerrillos	-32.628333	-70.940278	MF2	OP886940	BB	DD	BB	BB	AA	AA	CC
2361	*Liolaemus monticola*	Mina Cerrillos	-32.628333	-70.940278	MF2	OP886944	BB	DD	BB	BB	AA	AA	AA
2362	*Liolaemus monticola*	Mina Cerrillos	-32.628333	-70.940278	MF2	-	BB	DD	AB	BB	AA	AA	AA
2364	*Liolaemus monticola*	Mina Cerrillos	-32.628333	-70.940278	MF2	OP886939	AB	DD	BB	BB	AA	AA	AA
2365	*Liolaemus monticola*	Mina Cerrillos	-32.628333	-70.940278	MF2	-	BB	DD	AB	BB	AA	AA	AA
2366	*Liolaemus monticola*	Mina Cerrillos	-32.628333	-70.940278	MF2	-	BB	DD	BB	BB	AA	AA	AA
2367	*Liolaemus monticola*	Mina Cerrillos	-32.628333	-70.940278	MF2	OP886942	BB	DD	BB	BB	AA	EE	AA
2368	*Liolaemus monticola*	Mina Cerrillos	-32.628333	-70.940278	MF2	OP886941	BB	DD	AB	BB	AA	AA	AA
2373	*Liolaemus monticola*	Cabrería	-32.823611	-71.004722	MF2	-	BB	DD	AB	BB	AA	AA	CC
2374	*Liolaemus monticola*	Cabrería	-32.823611	-71.004722	MF2	OP886947	BB	DD	AA	BB	AA	AA	AC
2375	*Liolaemus monticola*	Cabrería	-32.823611	-71.004722	MF2	-	BB	DD	AB	BB	AA	AA	AC
2376	*Liolaemus monticola*	Cabrería	-32.823611	-71.004722	MF2	AY851708	BB	DD	AB	BB	AA	AE	CC
2377	*Liolaemus monticola*	Cabrería	-32.823611	-71.004722	MF2	OP886945	BB	DD	AB	BB	AA	AE	AA
2378	*Liolaemus monticola*	Cabrería	-32.823611	-71.004722	MF2	-	BB	DD	BB	BB	AA	AE	AA
2379	*Liolaemus monticola*	Cabrería	-32.823611	-71.004722	MF2	OP886946	BB	DD	BB	BB	AA	EE	AA
2382	*Liolaemus monticola*	Lontué	-35.166667	-71.166667	S	AY851741	AA	AA	AA	AA	AA	AA	AA
2383	*Liolaemus monticola*	Lontué	-35.166667	-71.166667	S	-	AA	AA	AA	AA	AA	AA	AA
2384	*Liolaemus monticola*	Lontué	-35.166667	-71.166667	S	AY851740	AA	AA	AA	AA	AA	AA	AA
2385	*Liolaemus monticola*	Lontué	-35.166667	-71.166667	S	-	AA	AA	AA	AA	AA	AA	AA
2386	*Liolaemus monticola*	Lontué	-35.166667	-71.166667	S	-	AA	AA	AA	AA	AA	AA	AA
2387	*Liolaemus monticola*	Lontué	-35.166667	-71.166667	S	-	AA	AA	AA	AA	AA	AA	AA
2388	*Liolaemus monticola*	Lontué	-35.166667	-71.166667	S	-	AA	AA	AA	AA	AA	AA	AA
2447	*Liolaemus monticola*	Saladillo	-32.916667	-70.166667	N	-	AA	AA	AB	BB	AA	AA	AA
2448	*Liolaemus monticola*	Saladillo	-32.916667	-70.166667	N	-	AA	AA	BB	BB	AA	AA	AA
2449	*Liolaemus monticola*	Saladillo	-32.916667	-70.166667	N	-	AA	AA	AB	BB	AA	AA	AA
2450	*Liolaemus monticola*	Saladillo	-32.916667	-70.166667	N	-	AA	AA	BB	BB	AA	AA	AA
2451	*Liolaemus monticola*	Saladillo	-32.916667	-70.166667	N	-	AA	AA	AB	BB	AA	AA	AA
2452	*Liolaemus monticola*	Saladillo	-32.916667	-70.166667	N	OP886937	AA	AA	AB	BB	AA	AA	AA
2453	*Liolaemus monticola*	Saladillo	-32.916667	-70.166667	N	AY851711	AA	AA	AA	BB	AA	AA	AA
2454	*Liolaemus monticola*	Saladillo	-32.916667	-70.166667	N	-	AA	AA	AA	BB	AA	AA	AA
2455	*Liolaemus monticola*	Saladillo	-32.916667	-70.166667	N	OP886938	AA	AA	BB	BB	AA	AA	AA
2456	*Liolaemus monticola*	Saladillo	-32.916667	-70.166667	N	-	AA	AA	BB	BB	AA	AA	AA
2457	*Liolaemus monticola*	Saladillo	-32.916667	-70.166667	N	-	AA	AA	AB	BB	AA	AA	AA
2458	*Liolaemus monticola*	Saladillo	-32.916667	-70.166667	N	-	AA	AA	AB	BB	AA	AA	AA
2459	*Liolaemus monticola*	Saladillo	-32.916667	-70.166667	N	-	AA	AA	BB	BB	AA	AA	AA
2460	*Liolaemus monticola*	Saladillo	-32.916667	-70.166667	N	-	AA	AA	AB	BB	AA	AA	AA
2463	*Liolaemus monticola*	Saladillo	-32.916667	-70.166667	N	-	AA	AA	AB	BB	AA	AA	AA
2465	*Liolaemus monticola*	Saladillo	-32.916667	-70.166667	N	-	AA	AA	AB	BB	AA	AA	AA
2466	*Liolaemus monticola*	Saladillo	-32.916667	-70.166667	N	-	AA	AA	BB	BB	AA	AA	AA
2467	*Liolaemus monticola*	Saladillo	-32.916667	-70.166667	N	-	AA	AA	AB	BB	AA	AA	AA
2468	*Liolaemus monticola*	Saladillo	-32.916667	-70.166667	N	-	AA	AA	BB	BB	AA	AA	AA
2474	*Liolaemus monticola*	Saladillo	-32.916667	-70.166667	N	-	AA	AA	BB	BB	AA	AA	AA
2475	*Liolaemus monticola*	Saladillo	-32.916667	-70.166667	N	-	AA	AA	BB	BB	AA	AA	AA
2508	*Liolaemus monticola*	Salamanca	-31.788889	-70.971111	NM2	OP886929	-	-	-	-	-	-	-
2509	*Liolaemus monticola*	Salamanca	-31.788889	-70.971111	NM2	OP886930	-	-	-	-	-	-	-
2511	*Liolaemus monticola*	Salamanca	-31.788889	-70.971111	NM2	OP886924	-	-	-	-	-	-	-
2539	*Liolaemus monticola*	Colorado Norte	-32.871111	-70.377778	NM1	-	AA	AA	BB	BB	AA	AA	AA

2540	*Liolaemus monticola*	Colorado Sur	-32.900278	-70.335556	NM1	-	AA	AA	AA	BB	AA	AA	AA
2541	*Liolaemus monticola*	Colorado Sur	-32.900278	-70.335556	NM1	-	AA	AA	BB	BB	AA	AE	AA
2542	*Liolaemus monticola*	Colorado Sur	-32.900278	-70.335556	NM1	-	AA	AA	BB	BB	AA	AA	AA
2543	*Liolaemus monticola*	Colorado Norte	-32.871111	-70.377778	NM1	OP886955	AA	AA	BB	BB	AA	EE	AA
2544	*Liolaemus monticola*	Colorado Norte	-32.871111	-70.377778	NM1	-	AA	AA	BB	BB	AA	AA	AC
2545	*Liolaemus monticola*	Colorado Norte	-32.871111	-70.377778	NM1	-	AA	AA	BB	BB	AA	AA	AC
2546	*Liolaemus monticola*	Colorado Norte	-32.871111	-70.377778	NM1	-	AA	AA	BB	BB	AA	AE	AC
2548	*Liolaemus monticola*	Colorado Sur	-32.900278	-70.335556	NM1	OP886917	AA	AA	BB	BB	AA	EE	AA
2549	*Liolaemus monticola*	Colorado Sur	-32.900278	-70.335556	NM1	-	AA	AA	BB	BB	AA	AE	AA
2550	*Liolaemus monticola*	Colorado Sur	-32.900278	-70.335556	NM1	-	AA	AA	BB	BB	AA	AA	CC
2551	*Liolaemus monticola*	Colorado Norte	-32.871111	-70.377778	NM1	AY851713	AA	AA	AB	BB	AA	AA	AC
2552	*Liolaemus monticola*	Colorado Norte	-32.871111	-70.377778	NM1	-	AA	AA	BB	BB	AA	AE	AA
2553	*Liolaemus monticola*	Colorado Norte	-32.871111	-70.377778	NM1	-	AA	AA	BB	BB	AA	AA	AC
2554	*Liolaemus monticola*	Colorado Norte	-32.871111	-70.377778	NM1	-	AA	AA	BB	BB	AA	AA	AA
2555	*Liolaemus monticola*	Colorado Norte	-32.871111	-70.377778	NM1	-	AA	AA	BB	BB	AA	AA	AC
2556	*Liolaemus monticola*	Colorado Norte	-32.871111	-70.377778	NM1	-	AA	AA	BB	BB	AA	AA	AC
2557	*Liolaemus monticola*	Colorado Norte	-32.871111	-70.377778	NM1	-	AA	AA	BB	BB	AA	AA	AA
2559	*Liolaemus monticola*	Colorado Sur	-32.900278	-70.335556	NM1	AY851714	AA	AA	AA	BB	AA	AA	AC
2560	*Liolaemus monticola*	Colorado Sur	-32.900278	-70.335556	NM1	-	AA	AA	BB	BB	AA	AA	CC
2561	*Liolaemus monticola*	Colorado Sur	-32.900278	-70.335556	NM1	-	AA	AA	AB	BB	AA	AA	AC
2562	*Liolaemus monticola*	Colorado Sur	-32.900278	-70.335556	NM1	-	AA	AA	BB	BB	AA	AA	AC
2564	*Liolaemus monticola*	Colorado Sur	-32.900278	-70.335556	NM1	OP886956	AA	AA	BB	BB	AA	AA	AA
2565	*Liolaemus monticola*	Colorado Sur	-32.900278	-70.335556	NM1	-	AA	AA	AB	BB	AA	AE	AA
2567	*Liolaemus monticola*	Colorado Sur	-32.900278	-70.335556	NM1	-	AA	AA	AB	BB	AA	AE	CC
2569	*Liolaemus monticola*	Colorado Sur	-32.900278	-70.335556	NM1	-	AA	AA	AA	BB	AA	AE	AA
2570	*Liolaemus monticola*	Colorado Sur	-32.900278	-70.335556	NM1	-	AA	AA	AB	BB	AA	AA	AA
2571	*Liolaemus monticola*	Colorado Sur	-32.900278	-70.335556	NM1	-	AA	AA	BB	BB	AA	AA	AA
2572	*Liolaemus monticola*	Colorado Sur	-32.900278	-70.335556	NM1	-	AA	AA	BB	BB	AA	AA	AA
2573	*Liolaemus monticola*	Colorado Sur	-32.900278	-70.335556	NM1	-	AA	AA	BB	BB	AA	AA	AA
2579	*Liolaemus monticola*	Colorado Sur	-32.900278	-70.335556	NM1	-	AA	AA	BB	BB	AA	AA	CC
2582	*Liolaemus monticola*	Colorado Sur	-32.900278	-70.335556	NM1	-	AA	AA	BB	BB	AA	AA	AC
2583	*Liolaemus monticola*	Los Queñes	-35.000000	-70.966667	S	-	AA	AA	AA	AA	AA	AA	AA
2584	*Liolaemus monticola*	Los Queñes	-35.000000	-70.966667	S	-	AA	AA	AA	AA	AA	AA	AA
2585	*Liolaemus monticola*	Los Queñes	-35.000000	-70.966667	S	-	AA	AA	AA	AA	AA	AA	AA
2613	*Liolaemus monticola*	Colorado Norte	-32.871111	-70.377778	NM1	-	AA	AA	BB	BB	AA	AA	AA
2614	*Liolaemus monticola*	Colorado Norte	-32.871111	-70.377778	NM1	-	AA	AA	AB	BB	AA	AA	AA
2615	*Liolaemus monticola*	Colorado Norte	-32.871111	-70.377778	NM1	-	AA	AA	BB	BB	AA	AA	AA
2616	*Liolaemus monticola*	Colorado Norte	-32.871111	-70.377778	NM1	-	AA	AA	BB	BB	AA	AE	AC
2617	*Liolaemus monticola*	Colorado Norte	-32.871111	-70.377778	NM1	-	AA	AA	BB	BB	AA	AE	AC
2619	*Liolaemus monticola*	Colorado Norte	-32.871111	-70.377778	NM1	-	AA	AA	AB	BB	AA	EE	CC
2620	*Liolaemus monticola*	Colorado Norte	-32.871111	-70.377778	NM1	-	AA	AA	BB	BB	AA	AA	AA
2622	*Liolaemus monticola*	Colorado Norte	-32.871111	-70.377778	NM1	-	AA	AA	AB	BB	AA	EE	AC
2623	*Liolaemus monticola*	Colorado Norte	-32.871111	-70.377778	NM1	-	AA	AA	AB	BB	AA	EE	AC
2638	*Liolaemus monticola*	Los Queñes	-35.000000	-70.966667	S	AY851739	-	-	-	-	-	-	-
2640	*Liolaemus monticola*	Los Queñes	-35.000000	-70.966667	S	-	AA	AA	AA	AA	AA	AA	AA
2642	*Liolaemus monticola*	Los Queñes	-35.000000	-70.966667	S	-	AA	AA	AA	AA	AA	AA	AA
2644	*Liolaemus monticola*	Los Queñes	-35.000000	-70.966667	S	-	AA	AA	AA	AA	AA	AA	AA
2655	*Liolaemus monticola*	Río Clarillo	-33.716667	-70.500000	S	AY851737	-	-	-	-	-	-	-
2661	*Liolaemus monticola*	Maipo Sur	-33.800000	-70.150000	S	AY851732	-	-	-	-	-	-	-
2665	*Liolaemus monticola*	Maipo Sur	-33.800000	-70.150000	S	AY851733	-	-	-	-	-	-	-
2688	*Liolaemus monticola*	La Dormida	-33.050000	-71.050000	N	AY851717	-	-	-	-	-	-	-
2759	*Liolaemus monticola*	Yeso Sur	-33.783333	-70.216667	S	AY851731	-	-	-	-	-	-	-
2762	*Liolaemus monticola*	Yeso Sur	-33.783333	-70.216667	S	AY851729	-	-	-	-	-	-	-
2763	*Liolaemus monticola*	Yeso Sur	-33.783333	-70.216667	S	AY851728	-	-	-	-	-	-	-
2783	*Liolaemus monticola*	Tilama	-32.072778	-71.218056	NM2	OP886919	-	-	-	-	-	-	-
2784	*Liolaemus monticola*	Tilama	-32.072778	-71.218056	NM2	OP886921	-	-	-	-	-	-	-
2794	*Liolaemus monticola*	El Roble	-32.966667	-71.000000	N	AY851716	-	-	-	-	-	-	-
2834	*Liolaemus monticola*	El Alfalfal	-33.533333	-70.266667	N	AY850616	-	-	-	-	-	-	-
2841	*Liolaemus monticola*	El Alfalfal	-33.533333	-70.266667	N	AY850615	-	-	-	-	-	-	-

2858	*Liolaemus monticola*	Yeso Norte	-33.783333	-70.200000	N	AY851722	-	-	-	-	-	-	-
2860	*Liolaemus monticola*	Yeso Norte	-33.783333	-70.200000	N	AY850614	-	-	-	-	-	-	-
2863	*Liolaemus monticola*	Los Patos	-32.508056	-70.575000	MF1	-	BB	DD	AB	BB	AA	-	-
2865	*Liolaemus monticola*	Los Patos	-32.508056	-70.575000	MF1	-	AB	DD	AB	BB	AA	AE	AA
2866	*Liolaemus monticola*	Los Patos	-32.508056	-70.575000	MF1	OP886964	BB	BD	BB	BB	AA	AE	AA
2867	*Liolaemus monticola*	Los Patos	-32.508056	-70.575000	MF1	OP886965	BB	BB	BB	BB	AA	AE	AA
2875	*Liolaemus monticola*	Rocín	-32.513611	-70.503611	MF1	OP886962	AB	DD	BB	BB	AA	AE	AA
2876	*Liolaemus monticola*	Chalaco	-32.501111	-70.582778	MF1	-	BB	DD	AB	BB	AA	AE	AA
2885	*Liolaemus monticola*	Chalaco	-32.501111	-70.582778	MF1	-	AB	BD	BB	BB	AA	AE	-
2886	*Liolaemus monticola*	Chalaco	-32.501111	-70.582778	MF1	-	AB	DD	BB	BB	AA	AE	AC
2887	*Liolaemus monticola*	Chalaco	-32.501111	-70.582778	MF1	-	BB	BD	BB	BB	AA	EE	AA
2888	*Liolaemus monticola*	Chalaco	-32.501111	-70.582778	MF1	-	AB	AD	BB	BB	AA	AA	AA
2889	*Liolaemus monticola*	Chalaco	-32.501111	-70.582778	MF1	OP886961	BB	BD	BB	BB	AA	AA	AA
2890	*Liolaemus monticola*	Los Patos	-32.508056	-70.575000	MF1	-	AB	DD	AB	BB	AA	AE	CC
2927	*Liolaemus monticola*	Tranquilla	-31.951667	-70.607222	NM2	OP886931	-	-	-	-	-	-	-
2928	*Liolaemus monticola*	Tranquilla	-31.951667	-70.607222	NM2	OP886934	-	-	-	-	-	-	-
2930	*Liolaemus monticola*	Tranquilla	-31.951667	-70.607222	NM2	OP886935	-	-	-	-	-	-	-
2931	*Liolaemus monticola*	Tranquilla	-31.951667	-70.607222	NM2	OP886933	-	-	-	-	-	-	-
2942	*Liolaemus monticola*	Tranquilla	-31.951667	-70.607222	NM2	OP886932	-	-	-	-	-	-	-
2952	*Liolaemus monticola*	Lo Vicuña	-32.595000	-70.740556	MF1	-	BB	BB	BB	BB	AA	AA	CC
2953	*Liolaemus monticola*	Lo Vicuña	-32.595000	-70.740556	MF1	-	BB	BD	AB	BB	AA	AE	AA
2955	*Liolaemus monticola*	Lo Vicuña	-32.595000	-70.740556	MF1	-	AB	DD	BB	BB	AA	EE	AA
2956	*Liolaemus monticola*	Lo Vicuña	-32.595000	-70.740556	MF1	-	BB	BD	AB	BB	AA	AA	AA
2958	*Liolaemus monticola*	Lo Vicuña	-32.595000	-70.740556	MF1	-	AB	DD	AB	BB	AA	AA	AA
2959	*Liolaemus monticola*	Los Patos	-32.508056	-70.575000	MF1	-	AB	BD	BB	BB	AA	AE	AC
2960	*Liolaemus monticola*	Los Patos	-32.508056	-70.575000	MF1	OP886958	BB	BD	BB	BB	AA	-	-
2961	*Liolaemus monticola*	Los Patos	-32.508056	-70.575000	MF1	-	AA	AD	BB	BB	AA	AE	AA
2962	*Liolaemus monticola*	Los Patos	-32.508056	-70.575000	MF1	OP886960	BB	BD	BB	BB	AA	-	AA
2963	*Liolaemus monticola*	Los Patos	-32.508056	-70.575000	MF1	-	AB	AD	AB	BB	AA	AA	AA
2964	*Liolaemus monticola*	Los Patos	-32.508056	-70.575000	MF1	-	AB	BD	AB	BB	AA	AE	AA
2967	*Liolaemus monticola*	Los Patos	-32.508056	-70.575000	MF1	-	BB	DD	BB	BB	AA	EE	AC
2970	*Liolaemus monticola*	Lo Vicuña	-32.595000	-70.740556	MF1	OP886966	BB	BD	AB	BB	AA	AE	-
2971	*Liolaemus monticola*	Lo Vicuña	-32.595000	-70.740556	MF1	-	BB	BD	AB	BB	AA	AA	AA
2972	*Liolaemus monticola*	Lo Vicuña	-32.595000	-70.740556	MF1	-	BB	DD	AB	BB	AA	AA	-
2975	*Liolaemus monticola*	Los Patos	-32.508056	-70.575000	MF1	OP886967	BB	DD	BB	BB	AA	AE	AA
2977	*Liolaemus monticola*	Los Patos	-32.508056	-70.575000	MF1	OP886959	BB	BD	BB	BB	AA	AE	CC
2990	*Liolaemus monticola*	Santa Virginia	-31.471944	-70.786111	NM2	OP886927	-	-	-	-	-	-	-
2991	*Liolaemus monticola*	Santa Virginia	-31.471944	-70.786111	NM2	OP886928	AA	AA	AF	AG	AA	AE	AA
2994	*Liolaemus monticola*	Illapel	-31.951944	-70.607222	NM2	OP886926	AA	AA	AF	AG	AA	AE	AA
3010	*Liolaemus monticola*	Chillepín	-31.824444	-70.860278	NM2	OP886925	-	-	-	-	-	-	-
3083	*Liolaemus monticola*	Chillepín	-31.824444	-70.860278	NM2	OP886923	-	-	-	-	-	-	-
3098	*Liolaemus monticola*	Salamanca	-31.788889	-70.971111	NM2	OP886922	-	-	-	-	-	-	-
3266	*Liolaemus monticola*	Colorado Sur	-32.900278	-70.335556	NM1	OP886936	-	-	-	-	-	-	-
3304	*Liolaemus monticola*	Siete Tazas	-35.166667	-71.166667	S	-	AA	AA	AA	AA	AA	AA	AA
3305	*Liolaemus monticola*	Siete Tazas	-35.166667	-71.166667	S	-	AA	AA	AA	AA	AA	AA	AA
3306	*Liolaemus monticola*	Siete Tazas	-35.166667	-71.166667	S	-	AA	AA	AA	AA	AA	AA	AA
3082	*Liolaemus fuscus*	Chillepín	-31.824444	-70.860278	-	MK472105	-	-	-	-	-	-	-

Supplementary Figures

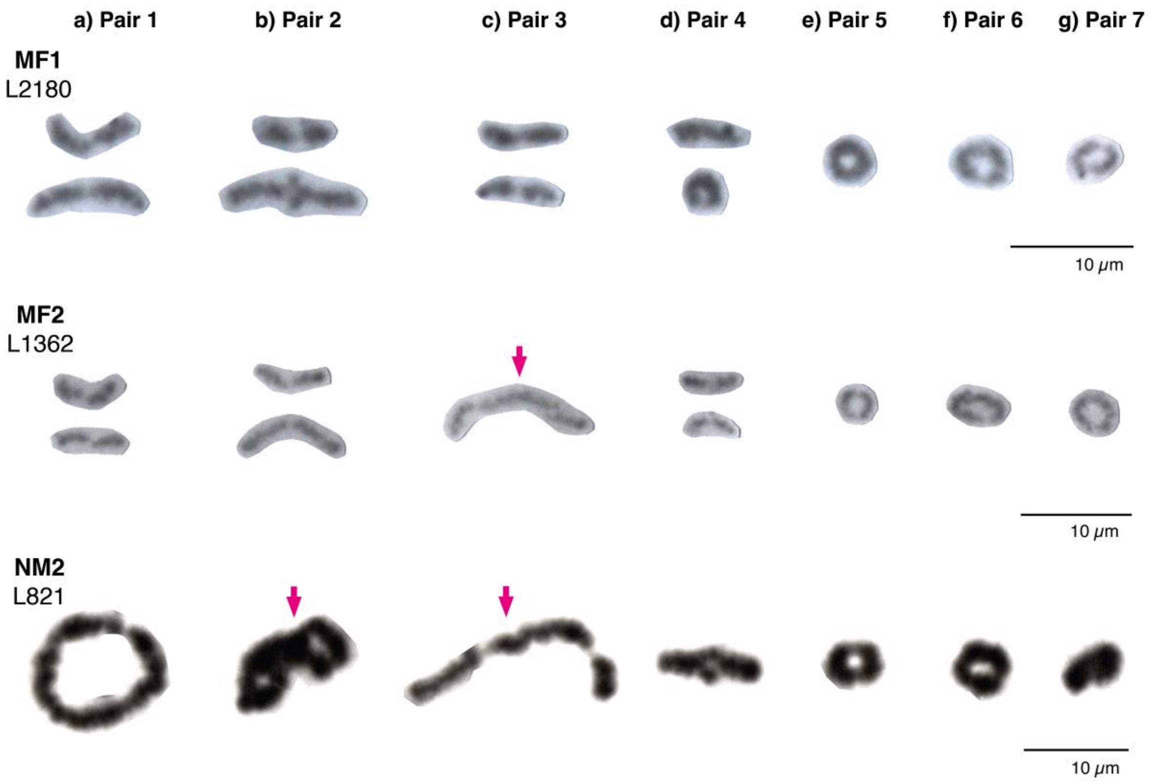

Figure S1. Representative meiotic karyotype for the first seven chromosome pairs for lizards from the new described chromosomal races of *Liolaemus monticola*. Arrows indicate pair 2 with 3 chiasmata in (b) and pair 3 trivalents in (c).

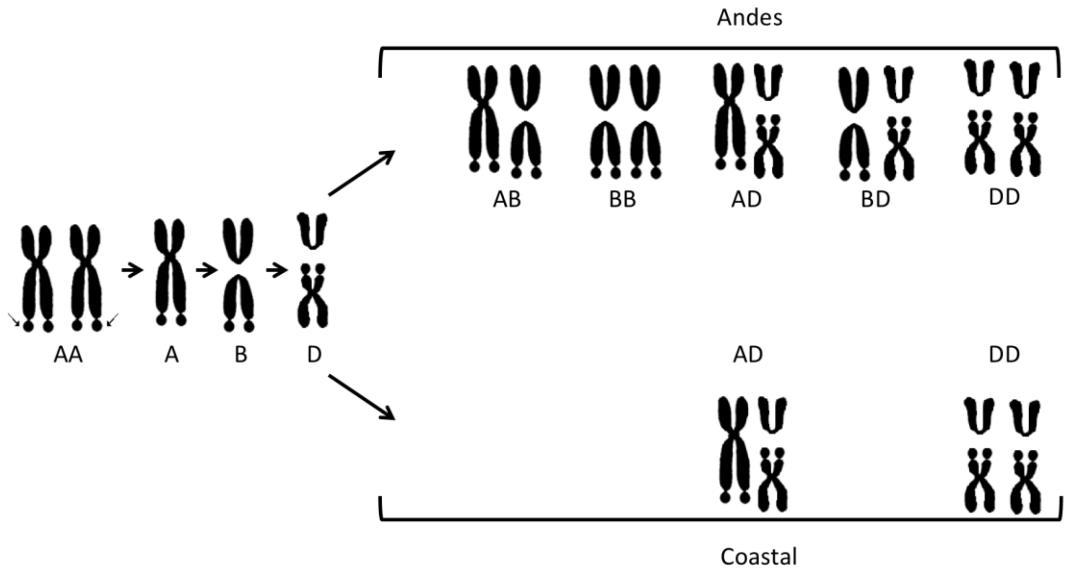

Figure S2. Model of chromosome pair 2 evolution for *Liolaemus monticola* in the Multiple Fission races. Note that in the Coastal range (MF2) the inversion product of pair 2 is fixated, whereas in the MF1 all the possible rearrangements occur.
